# Effects of Family-Based Interventions Using Mobile Apps on Youth’s Physical Activity: A Systematic Review

**DOI:** 10.3390/jcm11164798

**Published:** 2022-08-17

**Authors:** Pablo Rodríguez-González, Mohamed A. Hassan, Zan Gao

**Affiliations:** 1 School of Kinesiology, The University of Minnesota, Cooke Hall 208, 1900 University Ave. SE, Minneapolis, MN 55455, USA; 2 Faculty of Teacher Training and Education, University of Oviedo, 33006 Asturias, Spain; 3 Department of Methods and Curriculum, Physical Education College for Men, Helwan University, Cairo 12552, Egypt

**Keywords:** adolescents, children, eHealth, mHealth, smartphone

## Abstract

*Objective*. This review synthesized the currently available literature on the effects of family-based interventions using smartphone apps on youth physical activity. *Design*. Systematic review. *Data Sources*. 1037 studies from eight databases were retrieved. *Eligibility Criteria for Selecting Studies*. The seven articles included in this review met the following inclusion criteria: (1) experimental studies, (2) using smartphone apps, and (3) involving families with healthy children/adolescents. *Results*. Studies were stratified according to whether they used smartphone apps only or the combination of sports wearables and their associated companion app. The smartphone app interventions showed significant improvements in youth’s PA levels. All but one of the studies reported no significant improvement in PA levels after the intervention. However, positive PA-related outcomes were found, and the combination of sports wearables and their associated companion app showed inconclusive results due to the small number of studies. A trend of the relevance of families in improving the PA levels of youths was found. *Conclusions*. The findings of this review indicate that more research is needed on the effects of family-based interventions using mobile apps on youth’s physical activity. Mixed results were found for variables related to the PA of the youth involved in these programs. Although strong evidence was found that youth’s physical activity levels do not always improve with the implementation of these programs, promising results were found for a positive impact on different variables related to physical activity. Therefore, more experimental studies using only a mobile app to promote PA as the main outcome are needed to understand the real effect of mobile apps on youth’s PA levels. Future studies need to further explore this topic by developing programs based on designs of high methodological quality.

## 1. Introduction

After the declaration of the COVID-19 pandemic, many researchers intended to assess the consequences of isolation and quarantine [1]. Recent studies reported the prevalence of physical inactivity resulting from staying at home during the pandemic [2].The decrease in physical activity (PA) is not only leading to chronic disease such as cardiovascular health problems [3], but also it might be causing psychological disruptions such as anxiety and depression [4]. As a result, many studies summarized global recommendations referring to the crucial role of maintaining PA levels even while staying at home [5]. As a consequence, offering a variety of PA approaches became necessary. It is said that traditional PA approaches, such as school-based physical activities are offered on a limited basis due to the pandemic, therefore, other alternatives should be employed to promote PA.

One of the PA approaches is the use of recent technological means. However, there is a debate whether emerging technology is beneficial to promoting PA (e.g., individuals use health wearables to set and monitor PA goals) or it encourages sedentary behaviors (e.g., individuals engage in videogames) [6,7]. Thus far, over the past decade, research has supported the advantages of emerging technology. Numerous systematic reviews have reported the effectiveness of using different technologies in promoting PA behaviors and health outcomes among youth [8,9,10]. Simultaneously, technology devices (e.g., smartphones, and tablets) ownership has increased during the last few years [11,12]. This is conceivable considering that technology facilitates and speeds up many tasks, such as access to information or almost instantaneous communications with other people. Thus, the evolution of technological devices concerning PA approaches and applications is determined to be essential. As a consequence, research has already started to analyze the possibility of promoting health through mobile applications. Mollee et al. [13] analyzed features of the most recent applications used in the market and how they could influence PA promotion. Furthermore, they explained that using features such as social network sharing and user’s behavior input might be motivating to increase PA participation among users. Inversely, other studies have questioned the validity and reliability of the aforementioned applications and technological advances for use in PA interventions [14]. Thus, more research is needed to clarify the effects of PA programs using mobile apps. 

The effectiveness of mobile apps to promote PA has been previously analyzed by several systematic reviews in children and adolescents [15], young adults [16], and older adults [17]. These studies found potential, although still unclear due to the limited amount of supporting evidence to promote PA behaviors using mobile apps. Additionally, PA interventions comprising wearables and smartphone applications seem to be effective to promote PA [18,19]. The integration of health wearables (e.g., Fitbit) and smartphone apps has led to a large number of consumers using the wide range of functions that are accessible by novel mHealth (i.e., mobile health) applications to enhance the ability to track PA behaviors and records. However, it is still unclear to what extent the results can be attributed to the mobile app, sports wearable, or the combination when used together.

Numerous studies reported in the literature have investigated the effect of the use of technological devices to promote PA levels, but a limited number of supporting studies have evaluated the impact of parent and child involvement in mHealth-enhanced PA approaches. Nevertheless, some studies have reported the positive impact that a parent could exert to improve their child’s physical behaviors [20] and level of enjoyment [21]. Furthermore, a recent systematic review and meta-analysis that tested the effects of smartphone-based interventions on physical activity in children and adolescents reported that most of the studies included in their work required parental assistance for program implementation [22]. In this review, it was found that smartphone interventions have a significant effect on PA improvement and it was argued that parental assistance may have had a positive effect on this increase. In the same vein, Böhm et al. [15] concluded that a recommended design for interventions that include mobile health could be the combination of school-based interventions with family or community involvement. However, there is a gap in the literature on the effects of programs using mobile apps to promote physical activity in children within the family context. That is, it is unknown whether the results obtained by He et al. [22] for the set of smartphone-based interventions can also be applied to programs using mobile applications.

Therefore, the purpose of this study is to systematically evaluate the effect of smartphone app use on family-based interventions with the goal of promoting youth’s PA. The findings of this study could be beneficial in determining the effects of family-based interventions using mobile apps in children’s PA and the future of mobile applications that facilitate children and their parents and tailor more features to meet the expectations of families in PA settings.

## 2. Methods

The Preferred Reporting Items for Systematic Reviews and Meta-Analyses (PRISMA) [23] guidelines were followed in this systematic review.

### 2.1. Information Sources

A comprehensive systematic search of seven scientific literature databases (Web of Science, Academic Search Premier, Communication and Mass Media Complete, PubMed, Scopus, ERIC, and Medline) was conducted in April 2022.

### 2.2. Search Strategies

A total of 1037 studies were retrieved based on the combination of the following search descriptors: (“app” OR “mobile health” OR “mobile phone” OR “smartphone” OR “cell phone” OR “mHealth” OR “wearable activity monitor” OR “wearable activity tracker” OR “wearable device” OR “wearable technology” OR “wearable health technology”) AND (“family” OR “parent-targeted” OR “parent-focused” OR “parent-mediated” OR “parent-child” OR “parent-adolescent” OR “parent-led”).

### 2.3. Eligibility Criteria

Articles were included if they were published before April 2022. Table 1 displays the PICOTs (aka., population, intervention, comparator, outcomes, timing, and setting) and language eligibility criteria for this review. The criteria description and exclusion criteria are specified in the table. In addition, if several papers for the same experimental study were published, the most relevant one will be included. 

### 2.4. Data Collection Process

Three authors (PR-G, MH, ZG) established the search criteria, conducted the search on the databases, and retrieved the results. Two authors (PR-G, MH) independently analyzed the article records and selected those that met the inclusion criteria for further analysis, retrieved and reviewed the full articles, extracted data from them, and checked their bibliographies in search of new articles. The third author (ZG) supervised all these processes.

### 2.5. Data Items and Synthesis

Physical activity is the main outcome of interest for this review. MVPA and physical activity-related behaviors were included. The information collected was: purpose, sample and setting, data collection tools, design/method, and results. Each article was then assigned to one of the two categories (smartphone app, sports wearables plus companion app), and the results were evaluated based on the category to which each article belonged.

### 2.6. Risk of Bias in Individual Studies

Risk of bias for the individual studies included in this review was conducted using an 8-item scale. Each of the items was rated as positive (+), negative (−), or not applicable (NA), following previous literature [24,25,26]. Positive scores were given when the study clearly fulfilled the category. Negative scores were given when the category was missing or reported inaccurately. Not applicable was used for feasibility studies to which some categories do not apply. A final score was calculated by adding up all positive (+) scores. Studies were considered to have high design quality when these scores were five or more (median score), whereas studies receiving less than five were assessed as low quality with high risk of bias. 

### 2.7. Strength of Evidence

Based on a previously used evidence synthesis method [27], we evaluated the effects of interventions that analyzed the effect of the use of mobile apps in the family context on PA. Following the evaluation system adopted by Liang and Lau [28], and applied by Böhm et al. [15] to analyze the effect of mobile health to increase PA outcomes, five levels (strong, moderate, limited, inconclusive, and no effect) were defined based on study design and methodological quality. In addition, studies were stratified according to whether they used smartphone apps only or the combination of sports wearables and their associated companion app. According to Van Sluijs et al. [27], overall results are considered consistent if at least two-thirds of the relevant studies had significant results in the same direction.

## 3. Results

### 3.1. Study Selection

**Figure 1:**
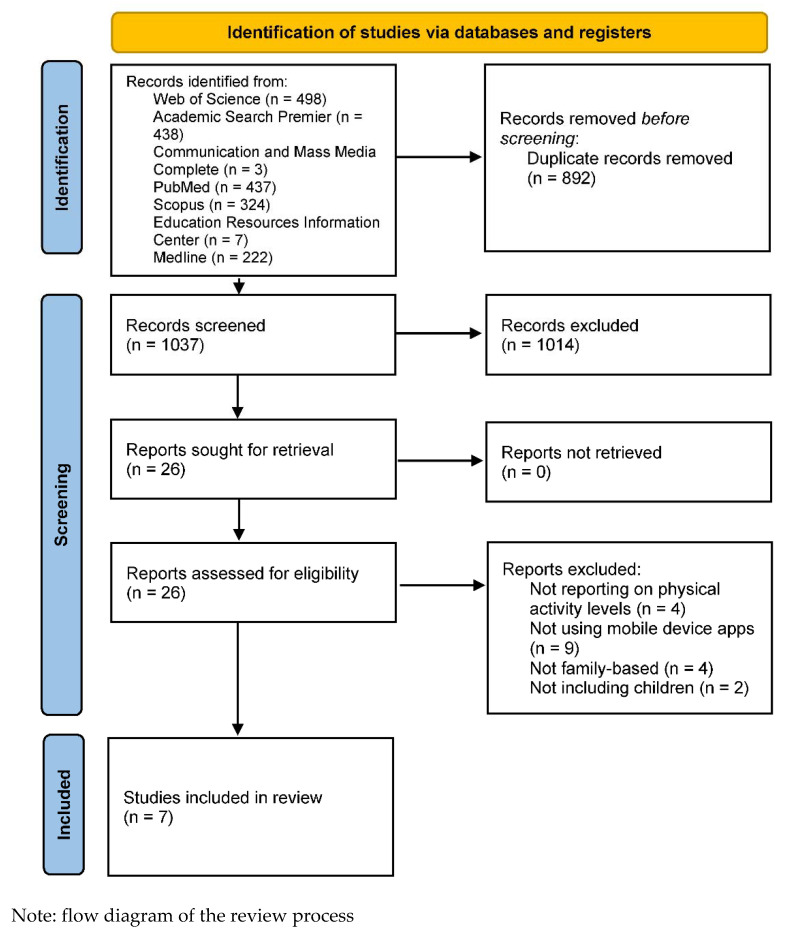
Flow Diagram of The Review.

After searching the databases, 1929 records were identified, of which 892 were eliminated before screening because they were duplicates. A total of 1037 studies were selected and 1014 were eliminated for failing to meet the inclusion criteria. After an in-depth evaluation of 26 full articles, seven studies were included in the review. In addition, after analyzing the references of the included articles, another article that met the inclusion criteria was found [29]. However, because the follow-up study for the same intervention was included [30], this article was not considered when writing the results. The flow diagram of this review is shown in Figure 1. Of these articles, four used a mobile app to promote PA, and three used a mobile app and sports wearable. Regarding study design, one randomized controlled trial [31], two feasibility studies with pre-post measures [32,33], one randomized clinical trial with pre-post measures [34], one pilot randomized trial [35], one follow-up study of a randomized controlled trial [30], and one pilot study with pre-post measures [36] were found. The articles included in this review were published between 2017 and 2022. Two experiments were conducted in each of the following countries: the United States, China, and Australia. One study was conducted in Sweden. Sample sizes ranged from nine dyads of parents and adolescents to 1392 children and their caregivers, and ages ranged from 3 to 17 years old. Finally, the duration of the intervention ranged from six weeks to 12 months. 

Moderate to vigorous physical activity (MVPA), achievement of active steps and minutes goals, PA behaviors, and the correlation between parent and child dropout from the program were evaluated by these studies. For this purpose, various measuring instruments were used. Accelerometers and activity trackers were predominantly used to assess PA levels. However, self-reported scales were also used for this purpose and to assess different behaviors related to PA.

### 3.2. Quality and Risk of Bias Assessment 

Table 2 reports the design quality analysis for the seven experimental studies included in this review. Overall, 85.7% of these studies [30,31,33,34,35,36] were of high methodological quality (5 or more points in the overall score). In addition, one (14%) of these studies obtained the highest possible score [30]. The lower scores in the risk of bias assessment are explained by the study design (feasibility studies). For these studies, randomization, control, baseline, and power analysis were not considered (Not Applicable).

### 3.3. Strength of Evidence

Three high-quality RCT studies that used mobile apps to promote youth’s PA in a family setting were included in this review [30,31,34]. The results showed strong evidence that these interventions did not result in a significant improvement in youth’s PA levels. However, positive PA-related outcomes were found in two of these RCTs [30,34]. In addition, a pilot study that found significant differences in children’s PA levels was included [36].

One high-quality RCT [35] and two feasibility studies [32,33] that used a sports wearable and its associated companion app were included. These studies analyzed variables related to PA. Phan et al. [35] found no significant differences between the control group and the experimental group. Schoeppe et al. [33] found significant differences in PA levels of children, fathers, and mothers. Bianchi-Hayes et al. [32] found that participants reached their step goals at least one-third of the time and their active minute goals more than half of the time. Therefore, the results cannot be considered consistent. 

### 3.4. Intervention Studies

#### 3.4.1. Mobile Apps

Five of the studies included in this review addressed the results of an intervention in which a mobile app was used to promote youth’s PA in a family setting [30,31,34,36]. The characteristics of included studies are shown in Table 3. The results showed mixed findings regarding significant improvements in youth’s PA levels. All of the studies, except Wong et al. [36], reported no significant improvement in PA levels after the intervention. However, positive PA-related outcomes were found. Liu et al. [34] developed a multifaceted program for obesity prevention in children in which the experimental group used a mobile app to strengthen family involvement. Youth in families that used the smartphone app during the program did significantly more PA with their parents than those who did not use it. Nyström et al. [30] conducted an RCT study to analyze the efficacy of a smartphone app-based obesity prevention program on PA, eating habits, and body fatness in healthy children. Children that received the intervention through a smartphone app (intervention group) had 99% higher odds of increasing a composite score for 6 dietary and PA variables. However, the effect observed after the 6-month intervention on the composite score was not maintained 6 months after it.

#### 3.4.2. Sports Wearables + Companion App

Three of the studies included in this review addressed the results of an intervention in which the combination of sports wearables and their associated companion app were used to promote youth’s PA in a family setting [32,33,35]. The findings were inconclusive. On one hand, Phan et al. [35] evaluated whether providing adolescents and their caregivers with a fitness tracker and its associated smartphone app could improve physical activity levels, among other variables. No significant differences in daily MVPA levels were found between the adolescent-only group and the adolescent-parent group. On the other hand, Schoeppe et al. [33] examined the feasibility and short-term effects of a six-week program to improve PA in families through a sports wearable and its associated smartphone app. Significant improvements were found in PA levels of children, fathers, and mothers. Finally, Bianchi-Hayes [32] implemented a ten-week feasibility and pilot study to promote physical activity in overweight or obese adolescents and their caregivers through a sports wearable and its associated app. Adolescents and their parents achieved the active minute goals more than half the time and the step goals at least one-third of the time. 

However, a trend of the relevance of families in improving the PA levels of youths was found. Phan et al. [35] had a high dropout rate (68%). As a consequence, it was possible to observe that adolescents were 12.2 times more likely to stop using the tracker if their parents did so earlier. In addition, Bianchi-Hayes et al. [32] found that the PA success rates of parents and adolescents were highly correlated.

## 4. Discussion

The main objective of this study was to review the current published literature evaluating the effects of family-based interventions using mobile apps on youth PA. A small number of studies (7) were found in this area, all of them published in the last five years (2017–2022). The studies took place in four countries: the United States (2), China (2), Sweden (1), and Australia (2). The majority of the studies implemented a program in which a mobile application was used (4/7), and the others implemented a program in which the combination of a sports wearable and its associated companion app was used (3/7). Overall, mixed results were found.

### 4.1. Mobile Apps

Among the studies that evaluated the effects of family-based interventions using a mobile app and targeting youth’s PA, three high-quality RCTs found no significant improvement in youth’s PA [30,31,34]. Conversely, a pilot study found significant differences in children’s PA levels [36]. Previous systematic reviews in adults found mixed results on MVPA levels after the intervention [18,19,37,38]. For instance, the most recent systematic review on this topic analyzed seventeen studies reporting results of smartphone-based interventions from which, eight studies revealed significant improvements in MVPA levels, while eight studies showed non-significant results, and one study resulted in a negative effect [24]. In children and adolescents, Böhm et al. [15] found seven studies using mobile health (smartphone app, text message) to improve physical activity outcomes in children and adolescents, including wearable activity trackers, with unclear conclusions. He et al. [22] conducted a systematic review and meta-analysis on the effects of smartphone-based interventions on physical activity in children and adolescents and found “promising” results for the use of these strategies to promote PA. Furthermore, apps were found to possibly be the most effective strategy. Therefore, the results of this review are in line with previous systematic reviews. 

In addition to the analysis of MVPA levels, some of the studies included in this review tested the effects of their programs on PA-related variables. Liu et al. [34] found that those participants whose family’s involvement was strengthened through a mobile app did significantly more PA with their parents than those who did not experience the program. Nyström et al. [30] reported that children who received the intervention through a smartphone app had 99% higher odds of increasing a composite score for the six dietary and PA variables assessed in the study. These results agree with previous systematic reviews on the promising effect of the use of interventions using mobile applications to promote PA. 

### 4.2. Sports Wearables + Companion App

Among the studies that evaluated the effects of family-based interventions using the combination of a sports wearable and its associated companion app to promote youth’s PA, one study found no significant improvements [35], and one found significant improvements not only for children but also for their caregivers [33]. The third study reported rates of achievement of minute and step goals for adolescents and their parents [32]. Therefore, due to the small number of studies, the mixed results, and the difference in age of the samples analyzed, the results are considered inconclusive. 

In accordance with what was stated by Xu et al. [39], where parents were found to play an important role in the support and management of child-related health behaviors, two of these studies reported findings on the relationship between parental and children involvement [32,33,34,35]. Due to a high dropout rate in Phan et al. [34], it was observed that adolescents were 12.2 times more likely to stop using the tracker if their parents did earlier. Additionally, Bianchi-Hayes et al. [32] found that PA success rates of parents and adolescents are highly correlated. These results support the idea that parents directly influence the healthy behaviors of young people. Therefore, the relevance of developing physical activity promotion programs in the family context is warranted.

### 4.3. Strengths and Limitations

This review has several strengths. First, to the best of our knowledge, this is the first systematic review analyzing the effects of family-based interventions using mobile apps on youth’s physical activity. Second, only true experimental studies were included, and thus the synthesized findings can be robust and convincing [40]. Third, the studies were divided and analyzed independently according to whether they used a mobile app or combination of sports wearables and their associated companion app. Lastly, the findings of this study provide a summary of the findings obtained so far by this type of app program, as well as theoretical and practical guidelines for the development of future app-based interventions.

However, the review has several limitations. First, due to the small number of included studies in this review and the mixed findings, no clear conclusions or recommendations can be offered. Second, most of the studies included in this review used a mobile app as an element of a comprehensive program. Therefore, it is unclear whether the results were derived from the use of a mobile app only or accompanied by other variables. Third, all of the studies addressed several outcomes, and some of them did not use PA as the primary outcome of the app interventions. Thus, the results could have been altered because of the aim of each intervention. 

## 5. Conclusions

The findings of this review indicate that more research is needed on the effects of family-based interventions using mobile apps on youth’s physical activity. Mixed results were found for variables related to the PA of youth involved in these programs. Furthermore, most of the studies used a mobile app as one component of a comprehensive design and focused on several outcomes. Thus, no clear conclusion can be drawn from this review. Future studies need to further explore this topic by developing app-based programs based on designs of high methodological quality. Although strong evidence was found that youth’s physical activity levels do not always improve with the implementation of these programs, promising results were found for a positive impact on different variables related to physical activity. Therefore, more experimental studies using only a mobile app to promote PA as the main outcome are needed to understand the real effect of mobile apps on youth’s PA levels. 

## Figures and Tables

**Table 1:** Population, Intervention, Comparator, Outcomes, Timing, and Setting (PICOTS) for Key Questions.

Inclusion Criteria	Criteria Description	Exclusion Criteria
Population	Families with children (parent-child)	
Intervention	Family-based interventions using smartphone apps to promote PA in children	
Comparison	Pre/post Control group	Non-experimental studies
Outcome	PA levels of children	
Timing	No restrictions	
Setting	No restrictions	
Language	English	Languages other than English

Note: inclusion and exclusion criteria for this review are presented.

**Table 2:** Design Quality Analysis.

Articles	(1) Randomization	(2) Control	(3) Pre-Post	(4) Retention	(5) Baseline	(6) Missing Data	(7) Power Analysis	(8) Validity Measure	(9) Six-Month Follow-Up	Score
Bianchi-Hayes et al. [31]	NA	NA	+	+	NA	−	NA	+	−	3
Liu et al. [33]	+	+	+	+	+	+	+	+	−	8
Nyström et al. [29]	+	+	+	+	+	+	+	+	+	8
Phan et al. [34]	+	−	−	−	+	+	+	+	−	5
Schoeppe et al. [32]	NA	NA	+	+	+	+	+	+	−	6
Trost et al. [30]	+	+	+	+	+	+	+	+	−	8
Wong et al. [35]	−	−	+	+	+	+	+	+	−	6

Note: (1) = randomization was performed and adequately explained; (2) = there was a control group and comparative analyses were performed between it and the intervention group; (3) = pre-post analyses were performed for outcome variables; (4) = dropouts did not exceed 30%; (5) = statistical differences were reported at baseline and groups were comparable on outcome variables; (6) = missing data were reported and considered for statistical analysis; (7) power analysis was performed; (8) validity and reliability of instruments were reported; (9) follow-up analysis was performed at 6 months or more. “+” refers to positive; “−” to negative; “NA” to not applicable. “+/−” represents significant improvements found in some measures while no significant effects were found in other measures.

**Table 3:** Synthesis of Studies to Generate a Literature Review.

Authors (Date)	Purpose and Variables	mHealth	Sample and Setting	Country	Data Collection Tools	Design/Method	Findings
Bianchi-Hayes et al. [31]	To evaluate the effect of a pilot study to promote PA for overweight and obese adolescents and their parents through smartphone-enabled (app) activity tracker data.	Sports wearable + associated companion app	9 parent-adolescent (14–16 years old) dyads. The adolescents were overweight or obese.	USA	A personal activity tracker (Jawbone UP MOVE) was used to count the number of steps taken per day. Pre and post surveys.	10-week intervention for adolescents and 1 parent using UP MOVE activity tracker and its mobile app. Single group (pre and post intervention surveys).	Both adolescents and their parents achieved step goals at least a third of the time and active-minutes goals more than half of the time. Both results were higher for parents. Parent-adolescent dyads have highly correlated PA success rates.
Liu et al. [33]	To test the effectiveness of a multifaceted intervention for obesity prevention in primary school children targeting children and their schools and families.	Smartphone app	1392 children (8–10 years old) and their caregivers; 670 in intervention, 703 in control.	China	PA (together with parents): an item self-reported by parents PA: updated version of a previously validated Youth Risk Behavior Survey questionnaire Physical fitness: one-minute rope jump, one-minute sit-up, long standing jump, shuttle run.	A school-year cluster randomized clinical trial pre and post intervention measures. The intervention schools experienced a multifaceted program, and the control group engaged in their usual practices. Family involvement was strengthened using a smartphone app.	PA behaviors improved. MVPA and physical fitness did not have significant changes.
Nyström et al. [29]	To analyze whether an intervention (MINISTOP) improved fat mass index and maintained the effect on a composite score consisting of FMI and dietary and PA variables 6 months after finishing it.	Smartphone app	315 children (4.5 years old) and their parents; 156 in the intervention, 159 in control.	Sweden	FMI and FFMI were calculated as kg divided m squared; Dietary patterns were assessed using the Tool for Energy Balance in Children during 4 days; PA was assessed through the ActiGraph wGT3x-BT	A 12-month follow-up study with Baseline, Midline, and post treatment measures was conducted for a previous RCT (Nyström et al., 2017). The intervention was delivered to the parents through a mobile app.	The intervention effect observed at the 6-month follow-up on the composite score was not maintained at the 12-month follow-up, with no effect on FMI being observed at either follow-up measure.
Phan et al. [34]	To explore whether providing caregivers and adolescents with a fitness tracker and its associated mobile app would improve PA levels, among others.	Sports wearable + associated companion app	88 adolescents (13–17 years old) who were new patients in a tertiary care weight management clinic and one caregiver for each were enrolled; 45 in the adolescent-only group, 43 in the dyad group.	USA	A fitness tracker was used to collect the number of steps, number of calories burned and number of MVPA minutes. The fitness tracker utility was assessed through Likert scales.	3-month pilot randomized trial. The participants were randomized to the adolescent-only group or the adolescent-parent group. All were provided a fitness tracker and its associated mobile app.	There were no significant differences between both groups for daily steps and daily MVPA. 69% of the adolescents reported that the fitness tracker helped them meet their goals and 66% that it motivated them towards achieving a healthy weight. However, 68% stopped using it during the study. In the dyad group, adolescents were 12.2 times more likely to stop using the tracker if their parents did it.
Schoeppe et al. [32]	To examine the feasibility and short-term effects of an intervention with an activity tracker and mobile app to increase PA in families (mothers, fathers and children).	Sports wearable + associated companion app	40 families from Queensland (Australia) composed of 58 children (6–10 years old), 33 fathers and 39 mothers.	Australia	An activity tracker (Garmin Vivofit) was used to assess the PA levels. Parent surveys were used to assess the effects (pre-post) of the intervention in the PA levels of both parents and children.	Single arm treatment with pre and post intervention measures. 6-week program based on individual and family goals, self-monitoring, performance feedback, family step challenges, family social support and modeling, weekly motivational text messages and an introductory session.	MVPA significantly increased by 58 min/day in children, 31 min/day in fathers and 27 min/day in mothers. Compliance with Australia’s PA guidelines increased from 34% to 89% in children, from 21% to 68% in fathers and from 8% to 57% in mothers. Families with at least one child and both parents meeting PA guidelines increased from 0% to 41%.
Trost et al. [30]	To evaluate the effectiveness of the Moovosity program, a novel digital application to increase FMS proficiency in 3- to 6-year-old children.	Smartphone app	34 parent-child () dyads; 17 in intervention, 17 in control.	Australia	Fundamental movement skills were assessed through the Test of Gross Motor Development. Children’s PA was assessed through a parent-reported checklist. Parental support for PA was measured through a 5-item scale.	8-week RCT with an intervention group receiving an app-based intervention and a waitlist-control group. Pre and post measures were evaluated.	The MoovosityTM app did not have a significant effect on children’s PA levels. Over the 8-week intervention period, PA levels in both the intervention and control group remained essentially unchanged.
Wong et al. [35]	To examine the effect of the Family Move app-based intervention on children’s health-related quality of life, psychosocial wellbeing, and PA levels.	Smartphone app	67 Chinese parent-child dyads took part.	China	PA was measured through the IPAQ questionnaire. Self-reported scales were used to assess health-related quality of life and psychosocial wellbeing. App usage was assessed based on the total of points earned.	8-week intervention using a mobile app. A 6-month follow-up was performed.	Children’s PA significantly increased during the intervention and post-intervention. Psychosocial outcomes declined 6 months after the start of the program. There was a low overall app usage.

Note: PA: physical activity; MVPA: moderate to vigorous physical activity; FMI: fat mass index; FFMI: fat-free mass index; RCT: randomized controlled trial; FMS: fundamental motor skills.

## Data Availability

The data used to support the findings of this study are available from the corresponding author upon request.
